# Low-Complexity Joint Angle of Arrival and Time of Arrival Estimation of Multipath Signal in UWB System

**DOI:** 10.3390/s23146363

**Published:** 2023-07-13

**Authors:** Weiming Deng, Jianfeng Li, Yawei Tang, Xiaofei Zhang

**Affiliations:** College of Electronic Information Engineering, Nanjing University of Aeronautics and Astronautics, Nanjing 211106, China; dengweiming@nuaa.edu.cn (W.D.); ywtang@nuaa.edu.cn (Y.T.); zhangxiaofei@nuaa.edu.cn (X.Z.)

**Keywords:** multipath environment, angle of arrival (AOA), time of arrival (TOA), multiple signal classification (MUSIC)

## Abstract

In an ultra-wideband (UWB) system, the two-dimensional (2D) multiple signal classification (MUSIC) algorithms based on high-precision 2D spectral peak search can jointly estimate the time of arrival (TOA) and angle of arrival (AOA). However, the computational complexity of 2D-MUSIC is very high, and the corresponding data model is only based on the dual antennas. To solve these problems, a low-complexity algorithm for joint AOA and TOA estimation of the multipath ultra-wideband signal is proposed. Firstly, the dual antenna sensing data model is extended to the antenna array case. Then, based on the array-sensing data model, the proposed algorithm transforms the 2D spectral peak search of 2D-MUSIC into a secondary optimization problem to extract the estimation of AOA via only 1D search. Finally, the acquired AOA estimations are brought back, and the TOA estimations are also obtained through a 1D search. Moreover, in the case of an unknown transmitted signal waveform, the proposed method can still distinguish the main path signal based on the time difference of arrival of different paths, which shows wider applications. The simulation results show that the proposed algorithm outperforms the Root-MUSIC algorithm and the estimation of signal parameters using the rotational invariance techniques (ESPRIT) algorithm, and keeps the same estimation accuracy but with greatly reduced computational complexity compared to the 2D-MUSIC algorithm.

## 1. Introduction

Angle of arrival (AOA) and time of arrival (TOA) are important issues in array signal processing, which is widely used in radar, navigation, indoor positioning, and wireless communication [1,2,3]. In the outdoor environment, the general positioning accuracy of the global positioning system (GPS) is more than 10 m without aids. GPS fails to help us in areas such as close-range complex following systems or indoor positioning. However, there are many applications in these complex environments, such as daily physical activity tracking [4], intelligent transportation [5], and localization of sensor nodes with a nano unmanned Aerial Vehicle [6]. In these close-range and complex scenarios, accurate estimation of AOA and TOA is important for positioning. AOA and TOA mean to measure the angle of arrival and time of arrival between the base station and the mobile station, respectively.

Ultra-wideband (UWB) signals [7] can solve the multipath problems to be faced in these scenarios. UWB signals are widely used in low-power short-range wireless communication [8] due to their large bandwidth, low power consumption, and strong ability to distinguish multipath signals. The Saleh-Valenzuela (S-V) model [9,10,11] has been adopted as a UWB system channel model for small-scale fading. In the S-V model, the multipath components are modeled as several rays arriving within different clusters. The multipath components of different clusters have constant AOA and TOA, but the fading is randomly varying. A series of algorithms are thus proposed to estimate AOA and TOA using the properties of the S-V channel model.

Earlier, only TOA in the UWB system was estimated, which was divided into time-domain methods, frequency-domain methods, and deep learning methods. Ref. [12] proposes a TOA estimation method based on matched filter threshold detection. Refs. [13,14] propose TOA estimation methods for incoherent energy detection based on a low sampling rate. A two-step TOA estimation method based on energy coherent detection of the direct path component is proposed in ref. [15]. The accuracy of the time-domain methods [12,13,14,15] is limited by the bandwidth and sampling interval. To meet the requirement of high resolution, TOA algorithms from the frequency domain [16,17,18] have been explored. Refs. [16,17,18] use a spectral peak search leading to an increase in computational complexity. Refs. [19,20,21] propose deep learning methods for TOA estimation. Ref. [19] proposes a machine learning method based on kernel principal component analysis, which projects selected channel parameters into a nonlinear orthogonal high-dimensional space and then uses a subset of these projections to estimate TOA. A deep learning model consisting of cascaded convolutional neural networks is proposed in ref. [20] to estimate TOA. Ref. [21] uses a deep learning model of ResNet50 [22] to estimate TOA. The deep learning methods reduce the computational complexity, but it is influenced by the data in the training set, which limits the applicability.

The AOA estimation in UWB systems has also been studied. Due to the high time resolution of ultra-wideband systems, a number of joint AOA and TOA estimation algorithms have been proposed. In [23], TOA is firstly estimated and then the best linear unbiased estimation is performed to obtain AOA. Ref. [24] improves the accuracy of TOA estimation by performing rough estimation and refining estimation, and the improvement of TOA estimation leads to the improvement of AOA estimation. In [25], a high-precision TOA estimation method is proposed based on two-dimensional multiple signal classification (2D-MUSIC), and the AOA can be obtained based on the time difference of adjacent array elements. Meanwhile, a Root-MUSIC algorithm is proposed to reduce the computational complexity in [25]. In [26], the computational complexity is further reduced compared with [25], which requires a spatial-spectral search to estimate the TOA. Ref. [26] estimates the TOA of two antennas using a polynomial root method, but the estimation accuracy is slightly reduced. Ref. [27] uses discrete Fourier transform (DFT) processing to obtain a coarse estimate of TOA, and designs a compensation matrix to phase compensate the time delay vector to obtain a fine estimate of TOA. The computational complexity of ref. [27] is greatly reduced because there is no need for eigenvalue decomposition, but the accuracy of TOA and AOA estimation is seriously affected by the number of DFT points. Ref. [28] exploits the conjugate symmetry of the time delay matrix to extend the sample points as well as the number of clusters to improve the estimation accuracy and the number of sources that can be identified. However, its computational complexity increases. In [29], the AOA is firstly estimated by frequency domain waveforms, and the TOA is estimated from AOA. Unlike [23,24,25,26,27,28], its AOA accuracy is not affected by the TOA estimation accuracy, but its TOA estimation accuracy is unsatisfied. Refs. [23,24,25,26,27,28,29] only consider dual antennas case, which does not consider the case of multiple antennas. In [30], the joint estimation of AOA and TOA is extended to three-dimensional space positioning using an iterative approach with high computational complexity, which is only applicable to the case of a single snapshot.

Although there are some works, e.g., refs. [23,24,25,26,27,28,29], considering the joint estimation of AOA and TOA in UWB system, only the data model of dual antennas is considered, which is not a general case. Meanwhile, the estimation accuracy and real-time performance of the methods in refs. [23,24,25,26,27,28,29,30] need to be improved. Moreover, in case of unknown prior information of the transmitted signal waveform, they are unable to distinguish the main path signal. To solve these problems, a low-complexity algorithm for joint AOA and TOA estimation of multipath signal in the UWB system is proposed. Firstly, the proposed algorithm transforms the 2D spectral search operation into a secondary optimization problem, and the AOA estimation is extracted separately. Then, the acquired AOA is brought into the 2D function, and the TOA estimation is also obtained through a 1D search. The main advantages of the proposed algorithm are as follows: (1) The proposed method has enhanced accuracy compared to the Root-MUSIC algorithm and ESPRIT algorithm, and maintains the same accuracy as the 2D-MUSIC algorithm; (2) Compared with the 2D-MUSIC, Root-MUSIC and EPRIT algorithm, the proposed algorithm requires lower complexity; (3) In case of unknown prior information of the transmitted signal, the proposed method can still distinguish the main path signal based on the time difference of arrival (TDOA) of a different path, which shows wider applications.

This paper is structured as follows: Section 2 introduces the received data model of the reference antenna and the proposed array-sensing data model. Section 3 introduces the traditional joint AOA and TOA estimation algorithms, including the 2D-MUSIC algorithm and the Root-MUSIC algorithm, respectively. Section 4 presents the proposed low-complexity joint AOA and TOA estimation algorithm. Section 5 shows the computational complexity and estimation accuracy of different algorithms, while conclusions are made in Section 6.

Notation: This article uses bold letters to denote vectors or matrices. ${\left(\right)}^{T}$, ${\left(\right)}^{*}$, ${\left(\right)}^{H}$, ${\left(\right)}^{-1}$ represent transpose, conjugate, conjugate transpose, and inverse of a matrix, respectively. $I_{N}$ represents the N×N identity matrix, and $E\left(\cdot \right)$ denotes expectation. ∧ denotes an estimated expression and ./ means point division of matrices. *∂* represents the partial derivative and $\left|\cdot \right|$ represents the absolute value. Finally, $diag\left(a\right)$ denotes the diagonal matrix with the elements of vector a as diagonal elements.

## 2. Data Model

### 2.1. Single-Antenna Sensing Data Model

**Figure 1:**
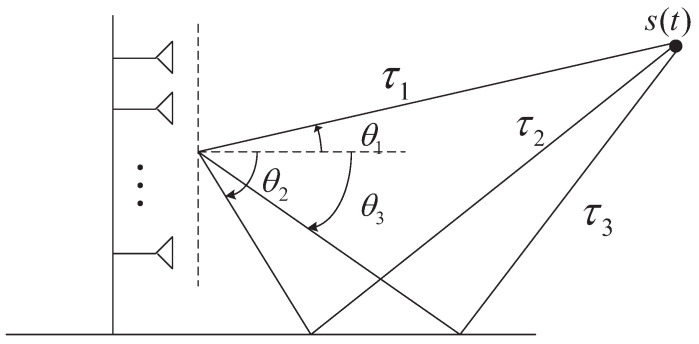
ULA structure for joint TOA and DOA estimation.

Figure 1 shows the scenario of multipath propagation and antenna array structure for joint AOA and TOA estimation. The antenna array is a uniform linear array (ULA), and the number of array elements is *M*. The array element spacing is d=λ/2, and λ is the wavelength corresponding to the center frequency of the incident signal. Assume that the incident signal propagating through the channel produces *L* multipath. The form of the signal received by the single antenna is denoted as

(1) $$y(t)=\sum_{l=1}^{L}\beta_{l}s\left(t-\tau_{l}\right)+w\left(t\right)$$

where s(t) is the transmitted signal, $\tau_{l}$ is the time delay value corresponding to each path, $\beta_{l}$ is the attenuation coefficient corresponding to each path, and w(t) is the zero-mean additive Gaussian white noise.

Then, we transform the time-domain form of the signal into the frequency-domain form as

(2) $$Y\left(\omega \right)=\sum_{l=1}^{L}\beta_{l}S\left(\omega \right)e^{-j\omega \tau_{l}}+W\left(\omega \right)$$

Sampling the received signal in the frequency-domain at equal intervals of *N*(N>L) points, with a sampling interval of Δω=2π/N, the discrete frequency-domain form of the sampled received signal can be obtained as follows:

(3) $$Y\left(\omega_{n}\right)=\sum_{l=1}^{L}\beta_{l}S\left(\omega_{n}\right)e^{-j\omega_{n}\tau_{l}}+W\left(\omega_{n}\right)$$

where $\omega_{n}=n\Delta \omega ,n=0,1,\cdots ,N-1$. Dividing the data into *K* segments (i.e., number of frequency-domain snapshots), the *k*th segment frequency-domain data can be written in the following concise vector form:

(4) $$y_{k}=SE_{\tau 1}\beta_{k}+w_{k}$$

where $y_{k}={\left[Y^{\left(k\right)}\left(\omega_{0}\right),\cdots ,Y^{\left(k\right)}\left(\omega_{N-1}\right)\right]}^{T}\in C^{N\times 1}$ is the *N*-point frequency-domain equal interval sampling of the received signal in the *k*th segment. $S=diag(\left[S\left(\omega_{0}\right),\cdots ,S\left(\omega_{N-1}\right)\right])$$\in C^{N\times N}$ is the diagonal matrix, and the diagonal elements are the *N*-point frequency-domain equal interval sampling values of the transmitted signal s(t). $w_{k}={\left[W^{\left(k\right)}\left(\omega_{0}\right),\cdots ,W^{\left(k\right)}\left(\omega_{N-1}\right)\right]}^{T}\in C^{N\times 1}$ is the frequency-domain sampling vector of Gaussian white noise. $E_{\tau 1}\in C^{N\times L}$ is the matrix containing the signal multipath time delay information. We refer to it as the time delay matrix. It can be expressed as $E_{\tau 1}=\left[e_{\tau_{1}},\;\cdots ,\;e_{\tau_{l}},\;\cdots ,\;e_{\tau_{L}}\right]$, where $e_{\tau_{l}}={\left[e^{-j\omega_{0}\tau_{l}},\;e^{-j\omega_{1}\tau_{l}},\;\cdots ,\;e^{-j\omega_{N-1}\tau_{l}}\right]}^{T}$. The coefficients of the fading channel are contained in the vector which can be expressed as $\beta_{k}=[\beta_{1}^{\left(k\right)},\;\beta_{2}^{\left(k\right)},\;\cdots ,\;\beta_{L}^{\left(k\right)}{]}^{T}$.

According to the S-V channel model, we can obtain the complex-value fading coefficient of the *k*th segment of the *l*th path as

(5) $$\beta_{l}^{\left(k\right)}=\alpha_{l}^{\left(k\right)}e^{j\phi_{l}^{\left(k\right)}}$$

where $\alpha_{l}^{\left(k\right)}$ is the fading amplitude of the *k*th segment of the *l*th path, and $\phi_{l}^{\left(k\right)}$ is an arbitrary phase variable representing a uniform distribution in the range $\left[0,2\pi \right]$. Through the S-V channel model, we can learn that $\beta_{l}^{\left(k\right)}$ is a random complex-value fading coefficient.

The current literature only considers the dual-antenna model, without considering the more generalized array antenna model. Here we propose the multiple array antenna elements receiving model.

### 2.2. Proposed Array Antenna Sensing Data Model

Previous methods have performed joint AOA and TOA estimation based on two receiving antennas, and have ignored the intrinsic connection of the delay matrix from each antenna. Here, we extend it to the case of multiple antennas. The frequency-domain received signal $Y_{i}\in C^{N\times K}\left(i=1,\cdots ,M\right)$ of each antenna can be obtained from the data model. It can be expressed as $Y_{i}=SE_{\tau i}B+W_{i}$, where $B=[\beta_{1},\cdots ,\beta_{k},\cdots ,\beta_{K}]\in C^{L\times K}$ represents the coefficient of the complex-value fading-channel, and $W_{i}=\left[w_{i1}^{},\cdots ,w_{ik}^{},\cdots ,w_{iK}^{}\right]\in C^{N\times K}$ denotes the frequency-domain sampling matrix of Gaussian white noise received by each antenna. $E_{\tau i}\in C^{N\times L}$ is the delay matrix of each antenna. The time delay matrix of the reference array element is expressed as

(6) $$E_{\tau 1}=\left[\begin{array}{cccc} e^{-j\omega_{0}\tau_{1}} & e^{-j\omega_{0}\tau_{2}} & \cdots & e^{-j\omega_{0}\tau_{L}}\\ e^{-j\omega_{1}\tau_{1}} & e^{-j\omega_{1}\tau_{2}} & \cdots & e^{-j\omega_{1}\tau_{L}}\\ \vdots & \vdots & \ddots & \vdots \\ e^{-j\omega_{N-1}\tau_{1}} & e^{-j\omega_{N-1}\tau_{2}} & \ldots & e^{-j\omega_{N-1}\tau_{L}} \end{array}\right]$$

The columns of the time delay matrix $E_{\tau i}$ of the *i*th antenna are related to the columns of the time delay matrix of the reference antenna as follows:

(7) $$e_{\tau il}=\Phi^{i-1}\left(\theta_{l}\right)e_{\tau 1l}$$

where $e_{\tau il}$ is the *l*th column of $E_{\tau i}$ and $\Phi \left(\theta_{l}\right)=diag\left(e^{-j\omega_{0}\frac{d\sin\theta_{l}}{c}},\;\ldots ,\;e^{-j\omega_{N-1}\frac{d\sin\theta_{l}}{c}}\right)$.

Because the general sampling bandwidth is larger than the signal bandwidth, many elements of $\left[S\left(\omega_{0}\right),\;\ldots ,\;S\left(\omega_{N-1}\right)\right]$ are zero or close to zero. Assuming non-zero at these frequencies in $\omega_{c0},\;\ldots ,\;\omega_{c(P-1)}$, the received signal matrix $Z\in C^{MP\times K}$ of *M* antenna array elements can be constructed as:

(8) $$\begin{array}{cc} Z & =\left[\begin{array}{c} Y_{1}\\ Y_{2}\\ \vdots \\ Y_{M} \end{array}\right]=\underset{A\left(\tau ,\theta \right)}{\underset{︸}{\left[\begin{array}{c} SF_{\tau 1}\\ SF_{\tau 2}\\ \vdots \\ SF_{\tau M} \end{array}\right]}}B+\underset{V}{\underset{︸}{\left[\begin{array}{c} V_{1}\\ V_{2}\\ \vdots \\ V_{M} \end{array}\right]}}\\ \; & =A\left(\tau ,\theta \right)B+V \end{array}$$

where $S=diag(\left[S\left(\omega_{c0}\right),\cdots ,S\left(\omega_{c(P-1)}\right)\right])$. $F_{\tau i}$ and $V_{i}$ are composed of rows of the matrix $E_{\tau i}$ and $W_{i}$ extracted according to $\omega_{c0},\;\ldots ,\;\omega_{c(P-1)}$, where $F_{\tau i}\in C^{P\times L}$ and $V_{i}\in C^{P\times K}$. τ is the time delay of the multipath signal arriving at the reference array, and θ is the angle of incidence of the multipath signal arriving at the array.

## 3. Traditional Based Joint AOA and TOA Algorithm

### 3.1. 2D-MUSIC Method

The covariance matrix of the received signal matrix Z can be written as follows:

(9) $${\hat{R}}_{ZZ}=ZZ^{H}/K$$

where ${\hat{R}}_{ZZ}\in C^{MP\times MP}$. The 2D-MUSIC [31] space spectral function is constructed as follows:

(10) $$P_{2D-MUSIC}\left(\tau ,\theta \right)=\frac{1}{a{\left(\tau ,\theta \right)}^{H}{\hat{U}}_{N}{\hat{U}}_{N}^{H}a\left(\tau ,\theta \right)}$$

where a(τ,θ) is the column vector in matrix A(τ,θ), and ${\hat{U}}_{N}\in C^{MP\times (MP-L)}$ represents the signal subspace composed of eigenvectors corresponding to the smaller MP−L eigenvalues. Let τ and θ vary and perform a 2D spectral peak search on Equation (10). $\hat{\tau }$ and $\hat{\theta }$ corresponding to the extreme spectral peak are the joint AOA and TOA estimates, where the shortest TOA corresponds to the main path signal.

### 3.2. Root-MUSIC Method

The method is a joint AOA and TOA estimation algorithm proposed in ref. [25] for dual-antenna. According to the previous data model, the reception data model of the dual-antenna can be obtained

(11) $$\begin{array}{c} Y_{1}=SE_{\tau 1}B+W_{1}\\ Y_{2}=SE_{\tau 2}B+W_{2} \end{array}$$

The received signal model of the dual antenna is

(12) $$Z=\left[\begin{array}{c} Y_{1}\\ Y_{2} \end{array}\right]=\left[\begin{array}{c} SE_{\tau 1}\\ SE_{\tau 2} \end{array}\right]B+\left[\begin{array}{c} W_{1}\\ W_{2} \end{array}\right]$$

where the TOA information contained in the time delay matrix $E_{\tau 1}$ of the first array element is $\tau_{l}\left(l=1,\;2,\;\ldots ,\;L\right)$. The TOA information contained in the time delay matrix $E_{\tau 2}$ of the second array element is $\varsigma_{l}\left(l=1,\;2,\;\ldots ,\;L\right)$.

The eigenvalue decomposition of the covariance matrix ${\hat{R}}_{1}=Y_{1}Y_{1}^{H}/K$ is performed to obtain the signal subspace and noise subspace.

(13) $${\hat{R}}_{1}={\hat{E}}_{S}{\hat{\Lambda }}_{S}{\hat{E}}_{S}^{H}+{\hat{E}}_{N}{\hat{\Lambda }}_{N}{\hat{E}}_{N}^{H}$$

where ${\hat{\Lambda }}_{S}$ is an L×L diagonal matrix whose diagonal elements contain *L* maximum eigenvalues. ${\hat{\Lambda }}_{N}$ represents the diagonal matrix whose diagonal elements contain N−L minimum eigenvalues. ${\hat{E}}_{S}$ is a matrix consisting of the eigenvectors corresponding to the *L* largest eigenvalues of ${\hat{R}}_{1}$. ${\hat{E}}_{N}$ stands for the matrix composed of the remaining eigenvectors. Then, we construct the one-dimensional spectral peak function as follows

(14) $$P_{MUSIC}\left(\tau \right)=\frac{1}{{\left[Sb(\tau )\right]}^{H}{\hat{E}}_{N}{\hat{E}}_{N}^{H}\left[Sb(\tau )\right]}$$

By utilizing the Root-MUSIC [32] idea, we use polynomial rooting instead of spectral peak search. Let $z=e^{-j\Delta \omega \tau }$, $p\left(z\right)=\left[1,z,\ldots ,z^{N-1}\right]$, and $p{\left(z\right)}^{H}=p{\left(z^{-1}\right)}^{T}=\left[1,z^{-1},\ldots ,{z^{}}^{-(N-1)}\right]$. Equation (14) is transformed into the following form

(15) $$\hat{f}\left(z\right)=z^{N-1}{\left[Sp\left(z\right)\right]}^{H}E_{N}E_{N}^{H}\left[Sp\left(z\right)\right]$$

Since the polynomial coefficients are symmetric, the roots are in the form of complex conjugate pairs, and the *L* roots ${\hat{z}}_{1},\;{\hat{z}}_{2},\;\ldots ,\;{\hat{z}}_{L}$ closest to the unit circle within the unit circle can be selected. Then the time delay of the signal received by the first antenna is obtained

(16) $${\hat{\tau }}_{l,\;0}=-angle\left({\hat{z}}_{l}\right)/\Delta \omega ,\;\left(l=1,\;2,\;\ldots ,\;L\right)$$

Let $\Delta {\hat{\tau }}_{l}={\hat{\varsigma }}_{l}-{\hat{\tau }}_{l}$, which is the time delay difference of the adjacent array elements of the *l*th multipath. From Figure 1, we can obtain $\Delta {\hat{\tau }}_{l}=\frac{d\sin\theta_{l}}{c}$. Exploiting the relationship between $\tau_{l}$ and $\varsigma_{l}$, we can obtain $\tau_{l}-\frac{d}{c}\leq \varsigma_{l}\leq \tau_{l}+\frac{d}{c}$. We perform eigenvalue decomposition of the covariance matrix $\hat{R}=ZZ^{H}/K$ to obtain the noise subspace ${\hat{U}}_{N}\in {}^{2N\times (2N-L)}$. $\varsigma_{l}$ can be estimated using the following one-dimensional spectral peak search function

(17) $${\hat{\varsigma }}_{l}=\underset{\varsigma \in \left[{\hat{\tau }}_{l,0}-\frac{d}{c},{\hat{\tau }}_{l,0}+\frac{d}{c}\right]}{\arg\max}\frac{1}{a{\left({\hat{\tau }}_{l,0},\varsigma \right)}^{H}{\hat{U}}_{N}{\hat{U}}_{N}^{H}a\left({\hat{\tau }}_{l,0},\varsigma \right)},\;l=1,\;2,\;\ldots ,\;L$$

The TOA value of the second antenna can be obtained by the local search of Equation (17). Then $\tau_{l}$ can be estimated via Equation (18) by locally searching $\tau_{l}$ within $\tau_{l}\in \left[{\hat{\tau }}_{l,0}-\Delta \tau ,{\hat{\tau }}_{l,0}\right.$$\left.+\Delta \tau \right]$, where is Δτ a small value.

(18) $${\hat{\tau }}_{l}=\underset{\tau \in \left[{\hat{\tau }}_{l,0}-\frac{d}{c},{\hat{\tau }}_{l,0}+\frac{d}{c}\right]}{\arg\max}\frac{1}{a{\left(\tau ,{\hat{\varsigma }}_{l}\right)}^{H}{\hat{U}}_{N}{\hat{U}}_{N}^{H}a\left(\tau ,{\hat{\varsigma }}_{l}\right)},l=1,2,\ldots ,L$$

Then we can obtain an estimate of the angle of incidence ${\hat{\theta }}_{l}=\arcsin\left(\frac{\Delta {\hat{\tau }}_{l}c}{d}\right),l=1,\;2,\;\ldots ,\;L$. So far, we have implemented the Root-MUSIC algorithm for joint AOA and TOA estimation in UWB systems.

## 4. Proposed Algorithm

### 4.1. Theoretical Derivation

The denominator of Equation (10) can be rewritten as:

(19) $$\begin{array}{c} \begin{array}{cc} f\left(\tau ,\theta \right) & =a{\left(\tau ,\theta \right)}^{H}{\hat{U}}_{N}{\hat{U}}_{N}^{H}a\left(\tau ,\theta \right)\\ \; & ={\left[Se_{\tau 1l}\right]}^{H}Q\left(\theta \right)\left[Se_{\tau 1l}\right] \end{array} \end{array}$$

(20) $$Q\left(\theta \right)={\left[\begin{array}{c} I\\ \Phi (\theta )\\ \vdots \\ \Phi^{M-1}\left(\theta \right) \end{array}\right]}^{H}U_{N}U_{N}^{H}\left[\begin{array}{c} I\\ \Phi (\theta )\\ \vdots \\ \Phi^{M-1}\left(\theta \right) \end{array}\right]$$

Then, Equation (19) can be written as follows:

(21) $$\begin{array}{c} \begin{array}{c} f\left(\tau ,\theta \right)={\left|S(\omega_{c0})\right|}^{2}\left(\frac{e^{-j\omega_{c0}\tau }}{S^{*}\left(\omega_{c0}\right)}{\left[Se_{\tau 1l}\right]}^{H}Q\left(\theta \right)*\left[Se_{\tau 1l}\right]\frac{e^{j\omega_{c0}\tau }}{S(\omega_{c0})}\right) \end{array} \end{array}$$

where $S^{*}\left(\omega_{c0}\right)$ represents the conjugate of $S(\omega_{c0})$. Since the constants do not affect the search of the spectral peak function, Equation (21) can be written as

(22) $$f\left(\tau ,\theta \right)=\frac{e^{-j\omega_{c0}\tau }}{S^{*}\left(\omega_{c0}\right)}{\left[Se_{\tau 1l}\right]}^{H}Q\left(\theta \right)\left[Se_{\tau 1l}\right]\frac{e^{j\omega_{c0}\tau }}{S(\omega_{c0})}$$

Define $e\left(\tau \right)=Se_{\tau 1l}\frac{e^{j\omega_{c0}\tau }}{S(\omega_{c0})}={\left[\begin{array}{cccc} 1 & \frac{S(\omega_{c1})}{S(\omega_{c0})}e^{-j\omega_{1}\tau } & \cdots & \frac{S(\omega_{c(P-1)})}{S(\omega_{c0})}e^{-j\omega_{\left(P-1\right)}\tau } \end{array}\right]}^{T}$, and rewrite Equation (22) as

(23) $$f\left(\tau ,\theta \right)=e{\left(\tau \right)}^{H}Q\left(\theta \right)e\left(\tau \right)$$

Equation (23) is an objective function, and considering the use of $e_{1}^{H}e\left(\tau \right)=1$ to eliminate the tame solution of $e\left(\tau \right)=0$, where $e_{1}={\left[1,0,\ldots ,0\right]}^{T}\in R^{P\times 1}$, Equation (23) can be reformulated as:

(24) $$\min_{\theta ,\tau }e{\left(\tau \right)}^{H}Q\left(\theta \right)e\left(\tau \right)\left|\right|s.t.e_{1}^{H}e\left(\tau \right)=1$$

The cost function is expressed as:

(25) $$L\left(\theta ,\tau \right)=e{\left(\tau \right)}^{H}Q\left(\theta \right)e\left(\tau \right)-\lambda \left(e_{1}^{H}e\left(\tau \right)-1\right)$$

where λ is a constant. Then, we give the derivative of L(θ,τ)

(26) $$\frac{\partial }{\partial e(\tau )}L\left(\theta ,\tau \right)=2Q\left(\theta \right)e\left(\tau \right)-\lambda e_{1}$$

According to Equation (26), we obtain $e\left(\tau \right)=\mu Q{\left(\theta \right)}^{-1}e_{1}$, where μ is a constant. Since $e_{1}^{H}e\left(\tau \right)=1$, combined with $e\left(\tau \right)=\mu Q{\left(\theta \right)}^{-1}e_{1}$, we obtain $\mu =1/e_{1}^{H}Q{\left(\theta \right)}^{-1}e_{1}$. We bring μ into the $e\left(\tau \right)=\mu Q{\left(\theta \right)}^{-1}e_{1}$ to obtain $\hat{e}\left(\tau \right)=\frac{Q{\left(\theta \right)}^{-1}e_{1}}{e_{1}^{H}Q{\left(\theta \right)}^{-1}e_{1}}$.

By bringing $\hat{e}\left(\tau \right)$ into $\min_{\theta }e{\left(\tau \right)}^{H}Q\left(\theta \right)e\left(\tau \right)$, we obtain the estimate ${\hat{\theta }}_{l}$(l=1,2,…L) as:

(27) $${\hat{\theta }}_{l}=\underset{\theta }{\arg\min}\frac{1}{e_{1}^{H}Q{\left(\theta \right)}^{-1}e_{1}}=\underset{\theta }{\arg\max}e_{1}^{H}Q{\left(\theta \right)}^{-1}e_{1}$$

The AOA is obtained by finding the angle corresponding to the peak of the (1, 1)th element of $Q{\left(\theta \right)}^{-1}$. Then, the acquired AOA is brought into the 2D function, and the TOA estimation is also obtained through a 1D search. The 2D-MUSIC space spectral function can be rewritten as:

(28) $$P_{1D-MUSIC}\left(\tau \right)=\frac{1}{a{\left(\tau ,\hat{\theta }\right)}^{H}U_{N}U_{N}^{H}a\left(\tau ,\hat{\theta }\right)}$$

The advantage of this proposed method is that the accuracy is close to that of the 2D search, and the computational complexity is greatly reduced compared to the 2D-MUSIC algorithm.

### 4.2. Separation of Multipath Signals under Unknown Waveforms

In addition, in case of unknown prior information of the transmitted signal, the proposed method can distinguish the main path signal based on TDOA. With the obtained incidence angle ${\hat{\theta }}_{l}$(l=1,2,…L) and $\hat{e}\left(\tau \right)=\frac{Q{\left(\theta \right)}^{-1}e_{1}}{e_{1}^{H}Q{\left(\theta \right)}^{-1}e_{1}}$, we obtain $\hat{e}\left(\tau_{l}\right)\;\left(l=1,2,\ldots ,L\right)\in C^{P\times 1}$ as:

(29) $$\hat{e}\left(\tau_{l}\right)={\left[\frac{S(\omega_{c0})}{S(\omega_{c0})}e^{-j\omega_{0}\tau_{l}},\ldots ,\frac{S(\omega_{c(P-1)})}{S(\omega_{c0})}e^{-j\omega_{\left(P-1\right)}\tau_{l}}\right]}^{T}$$

Let $\hat{e}\left(\tau_{l}\right)=\left[\begin{array}{c} e_{1l}\\ E_{Al} \end{array}\right]=\left[\begin{array}{c} E_{Bl}\\ e_{2l} \end{array}\right]$, where $e_{1l}$ and $e_{2l}$ are the first and last rows of $e(\tau_{l})$, respectively. $E_{Al}$ and $E_{Bl}$ are the rest. At this point, we can obtain

(30) $$E_{Al}./E_{Bl}={\left[\frac{S(\omega_{c1})}{S(\omega_{c0})}e^{-j\Delta \omega \tau_{l}},\ldots ,\frac{S(\omega_{c(P-1)})}{S(\omega_{c(P-2)})}e^{-j\Delta \omega \tau_{l}}\right]}^{T}$$

A simple example of judgment is given below. Suppose now that the incidence angles of all paths are obtained. At this point, we can obtain

(31) $$\begin{array}{c} \begin{array}{cc} Q_{1} & =E_{A1}./E_{B1}\\ \; & ={\left[\frac{S(\omega_{c1})}{S(\omega_{c0})}e^{-j\Delta \omega \tau_{1}},\ldots ,\frac{S(\omega_{c(P-1)})}{S(\omega_{c(P-2)})}e^{-j\Delta \omega \tau_{1}}\right]}^{T}\\ Q_{2} & =E_{A2}./E_{B2}\\ \; & ={\left[\frac{S(\omega_{c1})}{S(\omega_{c0})}e^{-j\Delta \omega \tau_{2}},\ldots ,\frac{S(\omega_{c(P-1)})}{S(\omega_{c(P-2)})}e^{-j\Delta \omega \tau_{2}}\right]}^{T}\\ \vdots \\ Q_{L} & =E_{AL}./E_{BL}\\ \; & ={\left[\frac{S(\omega_{c1})}{S(\omega_{c0})}e^{-j\Delta \omega \tau_{L}},\ldots ,\frac{S(\omega_{c(P-1)})}{S(\omega_{c(P-2)})}e^{-j\Delta \omega \tau_{L}}\right]}^{T} \end{array} \end{array}$$

(32) $$Q_{a}./Q_{b}={\left[e^{-j\Delta \omega (\tau_{a}-\tau_{b})},\ldots ,e^{-j\Delta \omega (\tau_{a}-\tau_{b})}\right]}^{T}\left(a,b\in l\right)$$

This way, the main path signal can be distinguished by observing the positive or negative TDOA between all paths without relying on the sampled value of the transmitted signal.

## 5. Computational Complexity and Estimation Performance

### 5.1. Computational Complexity

The computational complexity of the proposed algorithm is $O\{2M^{3}P^{3}+\left(K-L+N_{2}L\right)M^{2}P^{2}+N_{1}M^{2}P^{3}+N_{1}MP^{3}\;+\;\left(\left(N_{1}+N_{2}L\right)M^{2}-\left(N_{1}+N_{2}L\right)M+2N_{1}\right)P^{3}/2$ $+\;N_{2}LMP^{2}+N_{2}LMP\}$. The computational complexity of the 2D-MUSIC [26] algorithm is $O\{2M^{3}P^{3}+\left(K-L+N_{1}N_{2}\right)M^{2}P^{2}+N_{1}N_{2}MP^{2}+N_{1}N_{2}M\left(M-1\right)P^{3}/2+N_{1}N_{2}MP\}$. The computational complexity of the Root-MUSIC [25] algorithm is $O\{\left(10+\left(2N_{2}L+1\right)\left(M^{2}{\left(M+1\right)}^{2}1-4\right)/4\right)P^{3}+\left(L+K-7\right)P^{2}+2N_{2}L(\left(M-1\right)\left(M+2\right)/2$ $+\;M\left(M+1\right)\left(2M+1\right)\left(1-2L\right)/6-\left(1-2L\right))P^{2}\}$. The computational complexity of the Esprit [25] algorithm is $O\{\left(2+\left(N_{2}L+1\right)\left(M^{2}{\left(M+1\right)}^{2}-4\right)/4\right)P^{3}+\left(L-3+K\right)P^{2}+N_{2}L(\left(M-1\right)\left(M+2\right)/2$ $+\;M\left(M+1\right)\left(2M+1\right)\left(1-2L\right)/6-\left(1-L\right))P^{2}+\left(3-2L+3L^{2}\right)P+\left(2L^{3}-3L^{2}+L-1\right)\}$. Here, *M* is the number of array elements, *K* is the number of frequency domain snapshots, *L* is the number of multipath, *P* is the number of sampled points, $N_{1}$ is the number of angle search points, and $N_{2}$ is the number of time delay search points.

**Figure 2:**
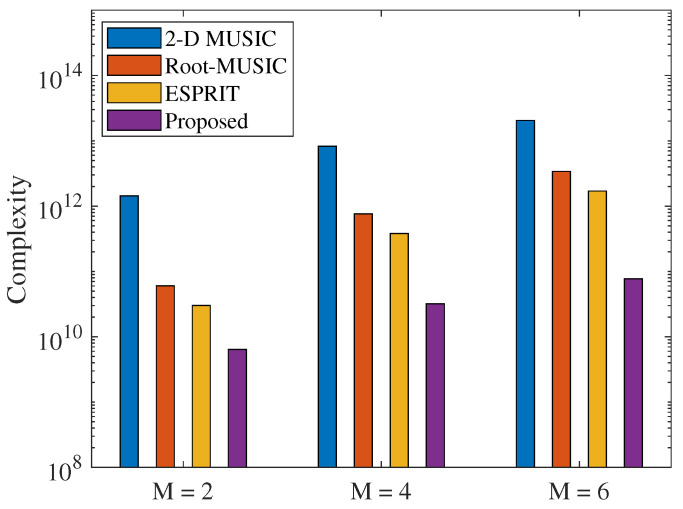
Computational complexity comparison.

Figure 2 shows a comparison of the computational complexity of the proposed method and the 2D-MUSIC algorithm with the variation of the number of array elements. With K=100, M=[2,4,6], L=3, P=64, $N_{1}=1001$, and $N_{2}=5001$. It can be seen from Figure 2 that the proposed method obtains lower computational complexity than other methods with different values of *M*. The computational complexity of the proposed algorithm is reduced by two orders of magnitude compared to the 2D-MUSIC and by one order of magnitude compared to the Esprit and Root-MUSIC. Thus, the proposed method provides an appreciable contribution to reducing the computational complexity, especially compared with other joint AOA and TOA estimation methods of the same type.

### 5.2. Simulation Parameters

To evaluate the performance of the proposed algorithm, we perform Monte Carlo simulations. The signal-to-noise ratio (SNR) and root mean square error (RMSE) [33] are defined, respectively, as

(33) $$\mathrm{SNR}=10\mathrm{lg}\frac{{∥y(t)∥}^{2}}{{∥w(t)∥}^{2}}$$

(34) $$\mathrm{RMSE}=\frac{1}{L}\sum_{l=1}^{L}\sqrt{\frac{1}{N}\sum_{n=1}^{N}{\left(\delta_{l}-{\hat{\delta }}_{l,n}\right)}^{2}}$$

where y(t) is the time-domain signal received by the antenna, w(t) is additive Gaussian white noise, *L* is the number of multipath, and *M* is the Monte Carlo count. ${\hat{\delta }}_{l,n}$ stands for the estimation of $\delta_{l}$ of the *n*th Monte Carlo trial. $\delta_{l}$ means the estimation of AOA and TOA.

**Figure 3:**
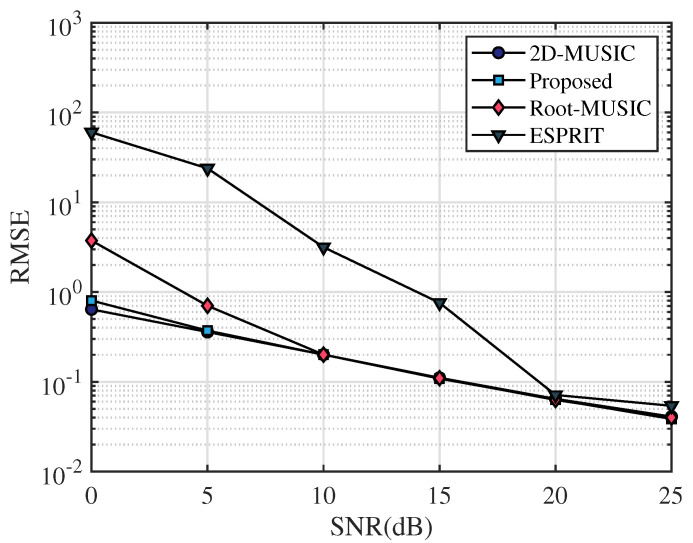
RMSE comparison of AOA under different SNR.

**Figure 4:**
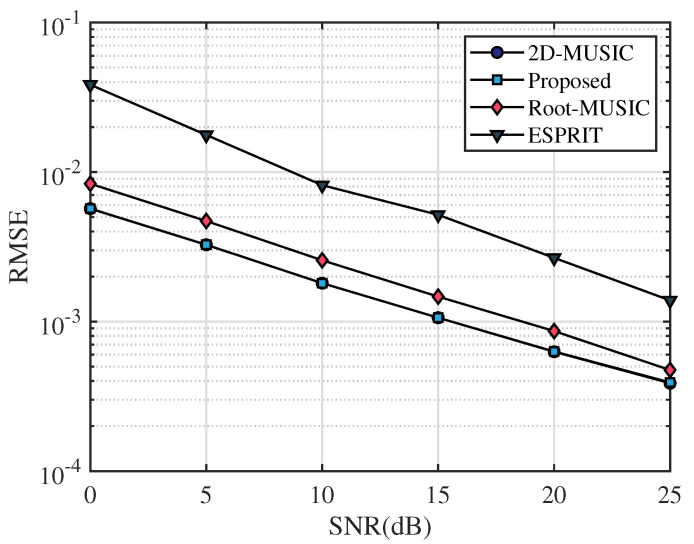
RMSE comparison of TOA under different SNR.

**Figure 5:**
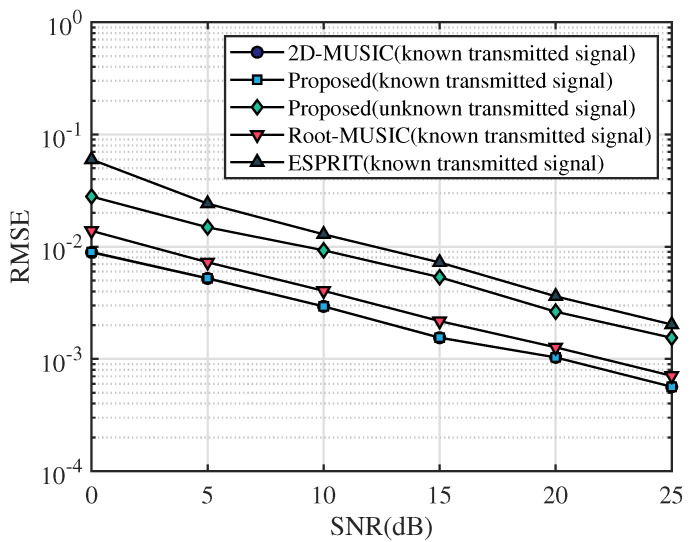
RMSE comparison of TDOA under different SNR.

**Figure 6:**
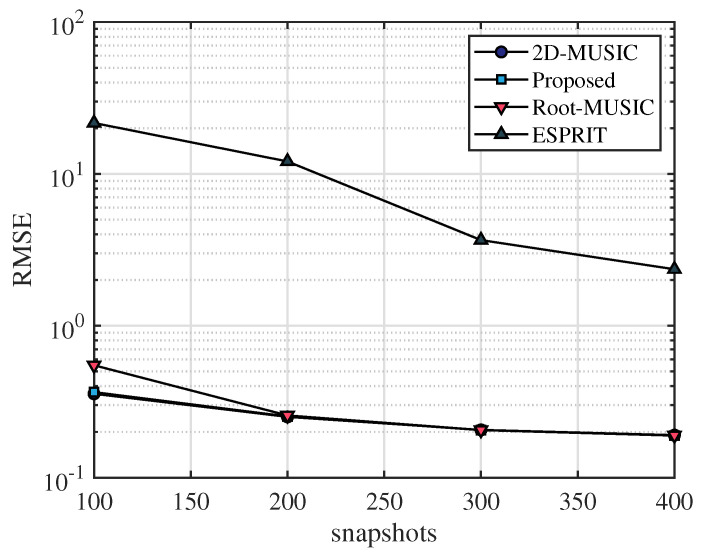
RMSE comparison of AOA under number of snapshots.

**Figure 7:**
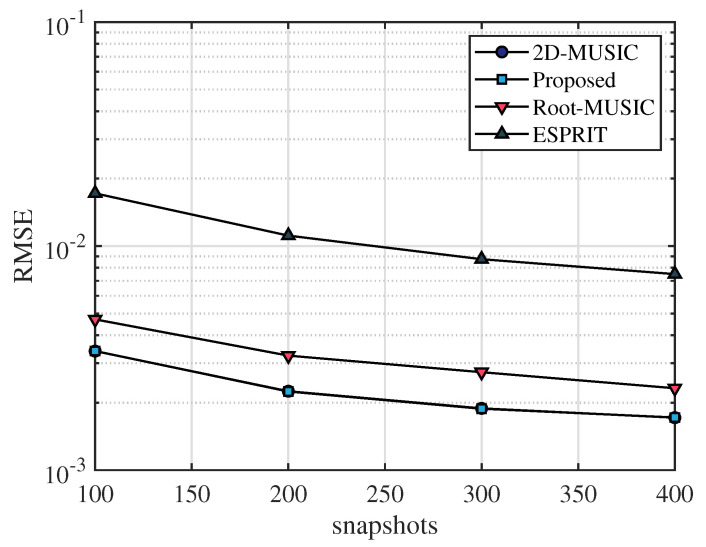
RMSE comparison of TOA under number of snapshots.

**Figure 8:**
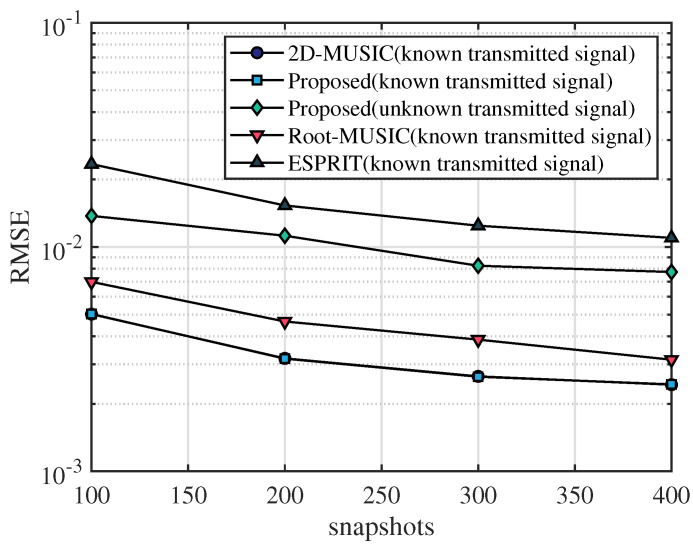
RMSE comparison of TDOA under number of snapshots.

In this section, the frequency range of the incident UWB signal is set from 3.5 GHz to 4.5 GHz, the bandwidth is 1 GHz, the sampling frequency is 10 GHz, and the number of sampling points is 2048. After that, we apply the fast Fourier transform on the sampled signal and select the number of points containing the signal as N=64. The frequency-domain snapshot number is K=100. The number of receiving antennas is M=2. We assume that the AOA of the multipath signal is $\left[20^{\circ },40^{\circ },60^{\circ }\right]$ and TOA of the multipath signal is [4.5/c,6.5/c,8.5/c], where *c* is the speed of light. Figure 3, Figure 4 and Figure 5 simulate the RMSE performance of AOA, TOA, and TDOA under different SNR varying from 0 dB to 25 dB. In Figure 5, except for the proposed algorithm (unknown transmitted signal) which estimates the TDOA with the unknown transmitted signal, all other algorithms estimate TDOA with the known transmitted signal. Figure 6, Figure 7 and Figure 8 simulate the RMSE performance of AOA, TOA, and TDOA at different snapshot numbers varying from K=100 to K=400 and SNR = 5 dB, respectively. In Figure 8, except for the proposed algorithm (unknown transmitted signal) which estimates the TDOA with the unknown transmitted signal, all other algorithms estimate TDOA with the known transmitted signal.

### 5.3. Results and Discussion

In this paper, the proposed algorithm is compared with the same type of joint AOA and TOA estimation algorithms, i.e., 2D-MUSIC in ref. [26], Root-MUSIC in ref. [25], and ESPRIT in ref. [25], respectively. From Figure 3 and Figure 4, it can be seen that the RMSE of AOA and TOA gradually decrease with the increase in the SNR. Meanwhile, it can be seen that the RMSE curves of AOA and TOA of the proposed algorithm basically overlap with those of 2D-MUSIC and are lower than Root-MUSIC and ESPRIT, indicating that the accuracy of AOA and TOA estimation of the proposed algorithm is basically consistent with 2D-MUSIC and better than Root-MUSIC and ESPRIT algorithms. Therefore, compared with other algorithms, the proposed algorithm has certain advantages in AOA and TOA estimation accuracy for the same SNR.

In order to show the ability of the proposed algorithm to discriminate the main path when the prior information of the transmitted signal is unknown, Figure 5 shows the TDOA estimation performance of the proposed algorithm in the case of unknown transmitted signal, and the rest of the compared algorithms estimate the TDOA in the case of known transmitted signal. The trend of the RMSE curve of the proposed algorithm (with known transmit signal) is similar to that of the RMSE curve of TOA shown in Figure 4. The accuracy of TDOA estimation for the proposed algorithm (known transmitted signal) is basically the same as 2D-MUSIC and better than Root-MUSIC and ESPRIT. In Figure 5, it can be seen that the TDOA estimation accuracy of the proposed algorithm (unknown transmitted signal) is better than the ESPRIT algorithm and slightly inferior to the Root-MUSIC algorithm, 2D-MUSIC algorithm, and the proposed algorithm (known transmitted signal). Although the TDOA estimation accuracy of the proposed algorithm (unknown transmitted signal) is general, it is applicable to a wide range of scenarios because it does not require a priori information of the known transmitted signal.

The number of snapshots is also an important factor affecting the estimation accuracy. In Figure 6, Figure 7 and Figure 8, simulation results show that the performance of RMSE improves as the number of snapshots increases. It can be seen in Figure 6 and Figure 7 that for the same number of snapshots, the AOA and TOA estimation accuracy of the proposed algorithm is basically the same as that of the 2D-MUSIC algorithm, which is better than other algorithms. It can be seen in Figure 8 that for the same number of snapshots, the TDOA estimation performance of the proposed algorithm (known transmitted signal) is basically the same as that of the 2D-MUSIC algorithm, and the TDOA performance of the proposed algorithm (unknown transmitted signal) is slightly inferior to that of the Root-MUSIC algorithm and better than that of the ESPRIT algorithm. Therefore, compared with other algorithms, the proposed algorithm has certain advantages in AOA, TOA, and TDOA estimation accuracy for the same number of snapshots, and the proposed algorithm is applicable to a wide range of scenarios.

## 6. Conclusions

In this paper, we proposed a high-precision and low-complexity joint AOA and TOA estimation algorithm. We extend the received data model of the dual antennas to the received data model of the multiple antennas. To reduce the computational complexity of the 2D-MUSIC joint AOA and TOA, the angular search can be extracted separately by converting the 2D spectral peak search into a secondary optimization problem. Then, the 2D search is converted into a 1D search based on the acquired AOA. As a result, the computational complexity is reduced, and the estimation accuracy of the proposed algorithm remains basically the same as that of the 2D-MUSIC algorithm. Meanwhile, in case of unknown prior information of the transmitted signal, the proposed method can distinguish the main path signal based on TDOA, which shows wider applications. Through simulation experiments, the following conclusions can be obtained:

(1) The computational efficiency of the proposed algorithm is significantly higher than that of the 2D-MUSIC algorithm, the Root-MUSIC algorithm, and the ESPRIT algorithm.
(2) At each SNR, the accuracy of AOA, TOA, and TDOA estimation of the proposed algorithm is basically the same as that of 2D-MUSIC and better than that of Root-MUSIC and ESPRIT.
(3) At each snapshot number, the accuracy of AOA, TOA, and TDOA estimation of the proposed algorithm is basically the same as that of 2D-MUSIC and better than that of Root-MUSIC and ESPRIT.
(4) In case of unknown prior information of the transmitted signal, the proposed method can still distinguish the main path signal, while the other methods fail.

The current methods can only handle a single signal. However, when multiple signals, especially those with multipath interference, are present, it becomes challenging to address the issue. Therefore, future research should focus on tackling the complexities associated with multiple signals. Additional investigation and exploration are necessary to thoroughly examine this aspect.

## Data Availability

Not applicable.

## Abbreviations

The following abbreviations are used in this manuscript:

AOA	Angle of Arrival
TOA	Time of Arrival
MUSIC	Multiple Signal Classification
2D	Two-Dimensional
Esprit	estimation of signal parameters using rotational invariance techniques
GPS	Global Positioning System
UWB	Ultra-Wideband
S-V	Saleh-Valenzuela
2D-MUSIC	Two-Dimensional Multiple Signal Classification
TDOA	Time Difference of Arrival
1D	One-Dimensional
ULA	Uniform Linear Array
SNR	Signal-to-Noise Ratio
RMSE	Root Mean Square Error
